# Homocysteine activates autophagy by inhibition of *CFTR* expression via interaction between DNA methylation and H3K27me3 in mouse liver

**DOI:** 10.1038/s41419-017-0216-z

**Published:** 2018-02-07

**Authors:** Anning Yang, Yun Jiao, Songhao Yang, Mei Deng, Xiaoling Yang, Caiyan Mao, Yue Sun, Ning Ding, Nan Li, Minghao Zhang, Shaoju Jin, Huiping Zhang, Yideng Jiang

**Affiliations:** 1 Department of Physiology and Pathophysiology, School of Basic Medical Sciences, Ningxia Medical University, Yinchuan, 750004 China; 2 Ningxia Key Laboratory of Vascular Injury and Repair Research, Yinchuan, China; 3 Ningxia Medical University General Hospital, Yinchuan, 750004 China; 4 Pharmacy college, Ningxia Medical University, Yinchuan, 750004 China

## Abstract

Elevated homocysteine (Hcy) levels have been reported to be involved in liver injury, and autophagy plays an important role in normal hepatic physiology and pathophysiology, but the mechanism underlying Hcy regulated autophagy is currently unknown. In this study, *CBS*^+/-^ mice were fed with regular diet for 12 weeks to establish a hyperhomocysteinemia (HHcy) model and HL-7702 cells were treated with Hcy, we found that Hcy increases autophagy and aggravates liver injury by downregulation of cystic fibrosis transmembrane conductance regulator (*CFTR*) expression in vivo and in vitro. Overexpression of *CFTR* inhibited the formation of autophagosomes and the expression of autophagy-related proteins BECN1, LC3-II/I and Atg12, while the expression of p62 increased in Hcy-treated hepatocytes and *CBS*^+/-^ mice injected with lentivirus expressing *CFTR*. Further study showed that *CFTR* expression is regulated by the interaction of DNA methyltransferase 1 (DNMT1) and enhancer of zeste homolog 2 (EZH2), which, respectively, regulate DNA methylation and histone H3 lysine 27 trimethylation (H3K27me3). In conclusion, our study showed that Hcy activates autophagy by inhibition of *CFTR* expression via interaction between H3K27me3 and DNA methylation in the mouse liver. These findings provide new insight into the mechanism of Hcy-induced autophagy in liver injury.

## Introduction

Homocysteine (Hcy) is a non-essential sulfhydryl-containing amino acid derived from methionine metabolism which takes place mainly in liver^1^. Dysregulation of methionine metabolism results in accumulation of Hcy in plasma, subsequently leading to liver injury^2,3^. Autophagy has emerged as a critical intracellular degradative pathway that maintains cell function and survival through the degradation of cellular components such as organelles and proteins^4^. Recent reports suggested that autophagy plays a role in liver homeostasis and energy conservation^5,6^; however, the underlying mechanism of Hcy regulated autophagy remain unclear.

The cystic fibrosis transmembrane conductance regulator (*CFTR*) is a cAMP-regulated chloride and bicarbonate channel that contributes to ion balance and fluid transport in a number of epithelial cell types^7,8^. There were evidences that *CFTR* regulates bile secretion and other functions at the apical membrane of biliary epithelial cells^9^. Loss of functional *CFTR* expression is thought to disturb the balance between fluid secretion and absorption into the epithelial layer, leading to cystic fibrosis liver disease^9,10^. Accordingly, a depressed autophagy has previously been reported in prostate cancer cells due to knockdown of *CFTR*^11,12^. Notably, liver is a major metabolic organ of Hcy, but there is no evidence to show the correlation between *CFTR* and hepatic autophagy induced by Hcy.

Epigenetic modifications such as DNA methylation and histone H3 lysine 27 methylation (H3K27me) can silence gene expression^13,14^. Hcy could act as a methyl donor during methylation of DNA and proteins^15^, growing evidence also suggests that Hcy may be involved in the interference of DNA methylation leading to the change of gene expression^16^. Our previous studies have found that Hcy could induce the hypomethylation of the FABP4 and upregulate its expression^17^. Furthermore, another important regulator of chromatin state is histone methylation, and which is catalyzed by a group of histone methyl transferases, such as EZH2, lysine 27 on histone H3 (H3K27me3)^18^. Some reports have shown that DZNep (an inhibitor of *S*-adenosyl-l-homocysteine hydrolase)-induced cell death results from the reduction of EZH2 expression due to its degradation by the proteasome, and in turn, from the reduction of the H3K27me3 expression^19,20^. Recently, the cross-talk between DNA methylation and H3K27me3 histone mark has been reported in a number of organisms and both marks are known to be required for proper developmental progression^21^. And the interaction between DNA methylation and H3K27me3 on gene regulation came into our sight as a potential goal worthy of further investigation.

In this study, we found that autophagy plays an important role in hepatic injury induced by Hcy. We also revealed that *CFTR* is an important regulator of autophagy, the expression of CTFR is regulated by interaction between H3K27me3 and DNA methylation, presenting evidence of new biomarkers for hepatic injury.

## Results

### Hcy induces autophagy in mouse liver and hepatocytes

**Fig. 1:**
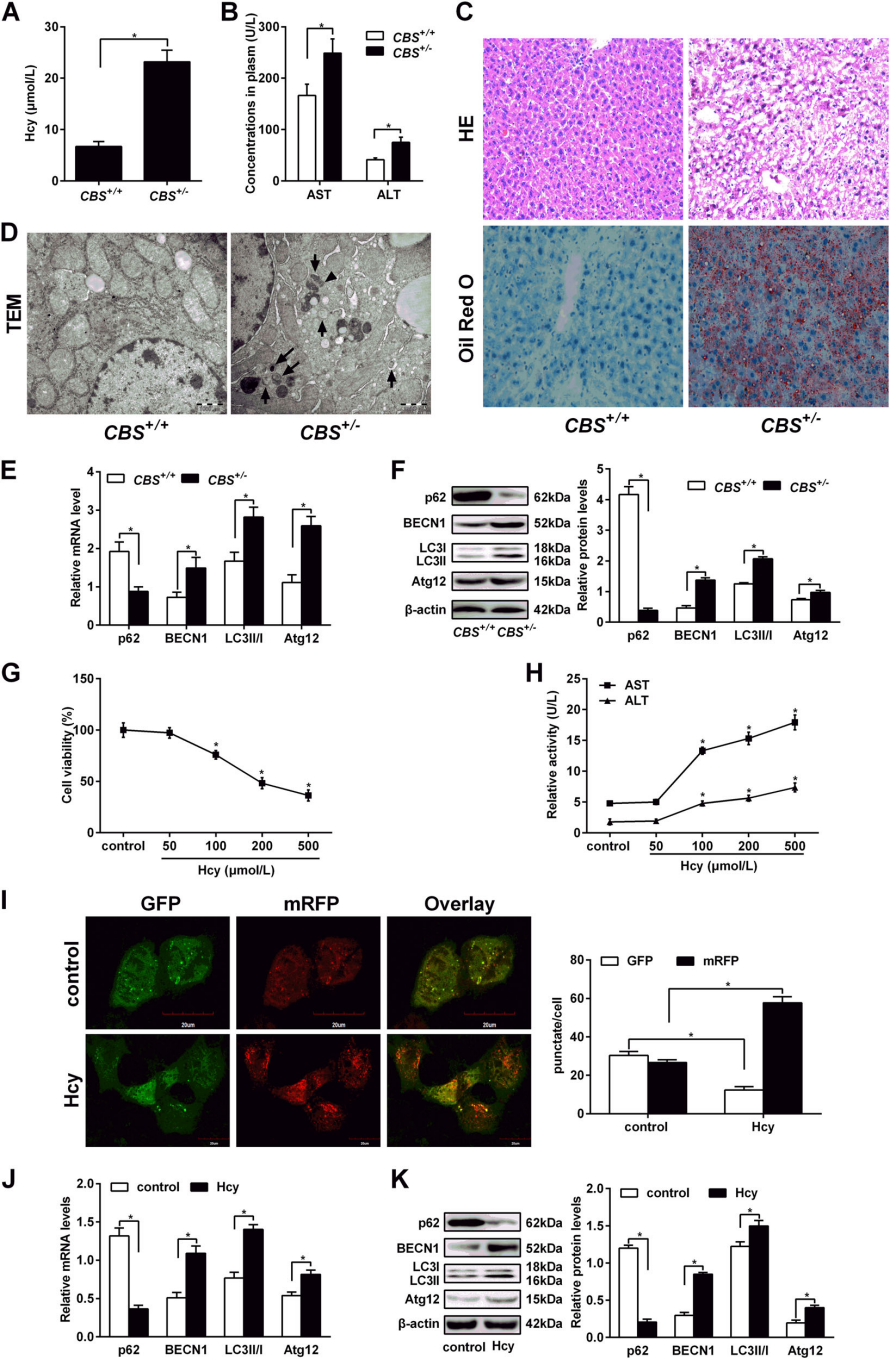
Hcy induces hepatic autophagy in *CBS*^+/-^ mice. Eight to 10 weeks old cystathionine b-synthase (*CBS*) heterozygous knockout mice (*CBS*^+/−^) were fed with regular mice chow and water ad libitum. Human hepatocytes HL-7702 were treated with L-Hcy (100 μmol/L) for 24 h. **a** The concentration of Hcy in plasma of mice was measured by automatic biochemical analyzer. **b** Contents of ALT and AST in serum of *CBS*^+/-^ mice were analyzed using automatic biochemical analyzer. **c** Hematoxylin and eosin (H&E) and Oil Red O staining of *CBS*^+/-^ mice liver. **d** Transmission electron microscope (TEM) was used to analyse cell autophagy in liver tissues of *CBS*^+/−^ mice. The arrows indicate the double-membrane vacuoles digesting organelles or cytosolic contents. **e** and **f** mRNA and protein expression of p62, BECN1, LC3 and Atg12 in the liver tissue of *CBS*^+/−^ mice by qRT-PCR and western blot, respectively. **g** Hepatocytes were treated with different concentrations of Hcy (50–500 μmol/L) for 24 h before MTT assay. **h** The activities of AST and ALT in hepatocytes after treatment with Hcy were detected by ELISA. (**I**) Confocal fluorescent microscopy analysis of hepatocytes overexpressing mRFP-GFP-LC3, treated with 100 µmol/L Hcy for 24 h. Quantification of mean red and green fluorescent puncta of at least 10 cells per condition is shown. The efficiency of transfection was also shown. (**J**) and (**K**) mRNA and protein expression of p62, BECN1, LC3 and Atg12 in hepatic cells treated with 100 µmol/L L-Hcy by qRT-PCR and western blot. Densitometry analysis of the proteins was performed for each sample (mean ± s.d.). ^*^P < 0.05

In order to know whether Hcy can induce hepatic autophagy, *CBS*^+/-^ mice were fed with a regular diet to establish HHcy animal model. Plasma Hcy levels in *CBS*^+/-^ mice were 3.46 times higher than that in *CBS*^+/+^ mice (Fig. 1a), indicating that hyperhomocysteinemia (HHcy) animal model was induced successfully. Next, we investigated the effects of Hcy on liver injury and found that the levels of serum indicators of liver function such as AST and ALT in *CBS*^+/−^ mice are much higher than that in *CBS*^+/+^ mice (Fig. 1a). Furthermore, the morphological changes of livers were assessed by H&E staining and Oil Red O. As shown in Fig. 1c, intact lobe structure with tidy hepatic cords and no degeneration, necrosis, or inflammatory cell infiltration were observed in *CBS*^+/+^ mice. Meanwhile, a considerable mass of fat vacuoles or deposition in hepatic tissue accompanied with mononuclear cells infiltration alongside the portal area and central vein was shown in liver of *CBS*^+/−^ mice, as well as ballooning and hydropic degeneration of hepatocytes. Oil Red O staining revealed much greater hepatic steatosis (fat deposition) in liver of *CBS*^+/-^ mice. Detection of *CBS*^+/-^ mice liver by transmission electron microscope (TEM) showed abundant autophagic vacuoles sequestrating cytoplasm and organelles, such as mitochondria and endoplasmic reticulum. Double-membranes, autophagosomes filled with degraded organelles and autolysosomes were frequently observed (Fig. 1d). Further detection of autophagy-related proteins expression showed that HHcy enhances the ratio of LC3-II/I and the expression of BECN1 and Atg12, and inhibits the expression of p62 in the *CBS*^+/-^mice (*P < *0.01, Fig. 1e, f). All these results suggest that HHcy induces liver autophagy in *CBS*^+/-^ mice.

To evaluate the most effective concentration of Hcy, hepatocytes were treated with Hcy at different concentrations (50–500 μmol/L) for 24 h. Cell death was significantly increased in Hcy (100–500 μmol/L)-treated cells after 24 h compared to untreated cells (Fig. 1g), and the activities of AST and ALT were upregulated in hepatocytes after treatment with Hcy in a concentration dependent manner (Fig. 1h). Notably, we found that 100 μmol/L of Hcy inhibits the cell viability to a minimal level, thus this dose was used for further experiments, as described below. Additionally, mRFP-GFP-LC3 adenoviral vectors were used to evaluate the autophagic level in cells treated with Hcy. Ad-mRFP-GFP-LC3, a specific marker for autophagosomes and autolysosomes, was transfected to hepatocytes. As expected, we found that cells treated with Hcy shows typically dense accumulation of mRFP-LC3 and GFP-LC3 puncta in the perinuclear region and cytoplasm. The numbers of mRFP-GFP-LC3 puncta dramatically increased in the cells upon Hcy treatment (Fig. 1i). Meanwhile, the ratio of LC3-II/I and the expression of BECN1 and Atg12 were upregulated and the expression of p62 was downregulated in hepatic cells treated with Hcy (Fig. 1j, k). The results suggested that Hcy induces autophagy of hepatic cells both in vivo and in vitro.

### Downregulation of *CFTR* plays a key role in Hcy induced hepatic autophagy

**Fig. 2:**
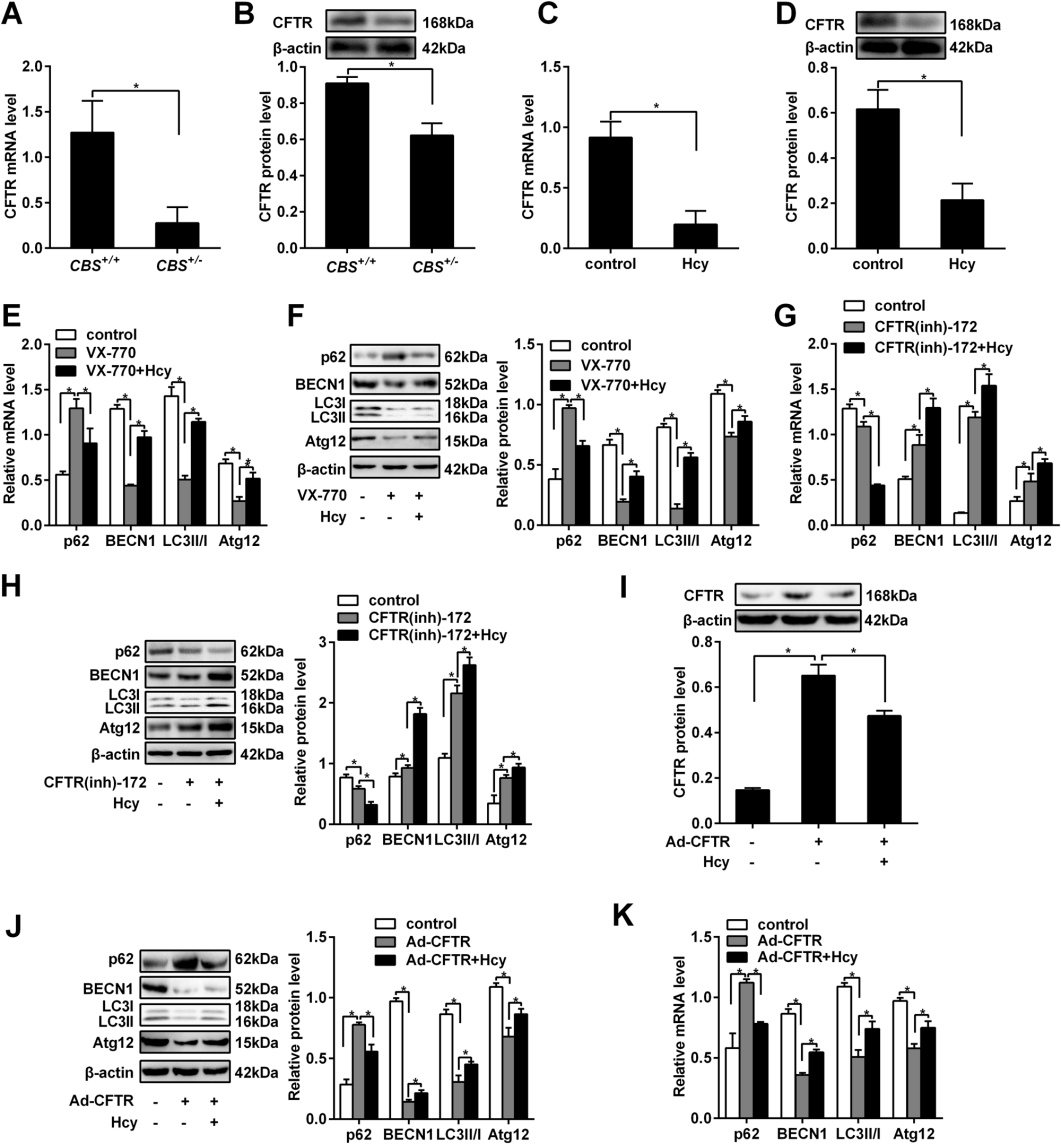
Hcy promotes the autophagy of hepatocytes by downregulation of cystic fibrosis trans membrane conductance regulator (*CFTR*). Eight to 10 weeks old cystathionine b-synthase (*CBS*) heterozygous knockout mice (*CBS*^+/−^) were fed with regular mice chow and water ad libitum. Human hepatocytes HL-7702 were treated with L-Hcy, VX-770 and CFTR(inh)-172, respectively, for 24 h. **a**,** b** qRT-PCR and western blot detected the mRNA and protein expression of *CFTR* in the liver of *CBS*^+/-^ mice. **c**, **d** qRT-PCR and western blot detected the mRNA and protein expression of *CFTR* in HL-7702 cells treated with 100 μmol/L Hcy. **e**,** f** Effect of *CFTR* activation on the expression of autophagy related proteins p62, BECN1, LC3-II/I and Atg12 in hepatocytes treated with VX-770. The mRNA and protein changes were detected by qRT-PCR and western blot respectively. **g**,** h** Effect of *CFTR* inhibition on the expression of autophagy related proteins p62, BECN1, LC3-II/I and Atg12 in hepatocytes treated with CFTR(inh)-172. The mRNA and protein changes were detected by qRT-PCR and western blot respectively. **i**,** j, k** An adenoviral vector carrying either a scrambled sequence or CFTR plasmid infected hepatocytes and protein expression of *CFTR* was detected by western blot, and the effect of *CFTR* overexpression and activation on the ratio of LC3-II/I and the expression of autophagy related proteins p62, BECN1 and Atg12 was examined in hepatocytes infected with Ad-CFTR. The mRNA and protein changes were detected by qRT-PCR and western blot respectively. Results represent the means ± s.d. of independent experiments. * *P* < 0.05

For verifying whether *CFTR* is involved in liver autophagy, *CFTR* mRNA and protein expression were analyzed by qRT-PCR and western blot in liver of *CBS*^+/-^ mice and hepatic cells treated with Hcy. We found that *CFTR* mRNA and protein expression decreased both in vivo and in vitro (*P < *0.05, Fig. 2a–d). To further explore the role of *CFTR* in autophagy, the hepatocytes were treated with an potentiator, ivacaftor (VX-770) or antagonist (CFTR(inh)-172), the results showed that the ratio of LC3-II/I and the expression levels of autophagy-related proteins BECN1 and Atg12 decreased and p62 increased in hepatocytes treated with VX-770. The effects of VX-770 are ameliorated (*P < *0.05, Fig. 2e, f) when hepatocytes were treated with Hcy. In contrast, the ratio of LC3-II/I and the expression levels of BECN1 and Atg12 increased and p62 decreased in hepatocytes treated with CFTR(inh)-172. The effects of CFTR(inh)-172 were enhanced when hepatocytes were treated with Hcy (*P < *0.05, Fig. 2g, h). Furthermore, to identify the effects, an adenoviral vector was constructed to overexpress *CFTR* in hepatocytes. *CFTR* protein expression was upregulated compared to the hepatocytes infected with adenoviral vector carrying a scrambled sequence. And on this basis, Hcy could downregulate the expression of *CFTR* in the hepatic cells (*P < *0.05, Fig. 2i). To test the effects of overexpression of *CFTR* in hepatocytes on the expression of autophagy-related proteins p62, BECN1, LC3, and Atg12, we detected their expression after infection. The results are similar as the treatment with the agonist of *CFTR*, VX-770 (*P < *0.05, Fig. 2j, k). This implies that Hcy treatment may repress *CFTR* transcription in the liver tissue of *CBS*^+/-^ mice and hepatocytes treated with Hcy.

### Augmentation of *CFTR* expression in the liver by in vivo lentivirus administrion reduces hepatic autophagy and liver injury in *CBS*^+/-^ mice

**Fig. 3:**
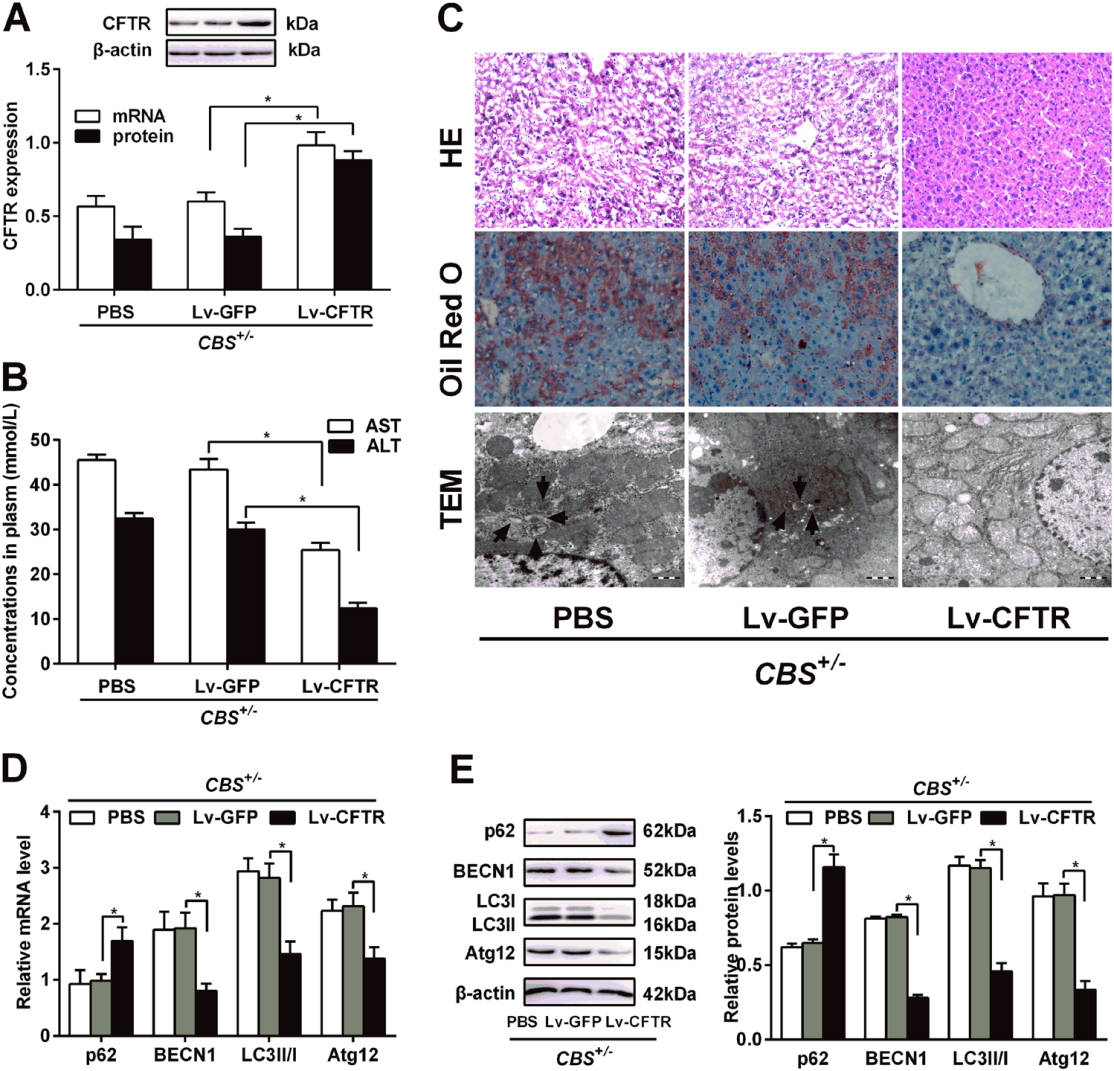
*CFTR* alters the levels of liver injury biomarker and autophagy in *CBS*^+/**−**^ mice. *CBS*^+/−^ mice injected with lentivirus Lv-CFTR or Lv-GFP through the tail vein (see Materials and Methods). **a** *CFTR* mRNA and protein levels were determined by qRT-PCR and western blot respectively. Data are presented as mean ± s.d. relative to Lv-GFP-injected mice. **b** Circulating levels of ALT and AST were analyzed. Results are means ± s.d. (*n* = 6). *P < 0.05. **c** Liver section from mice were subjected to H&E (upper) and Oil Red O (middle) staining. Transmission electron microscope (TEM) (under) was used to analyse cell autophagy in liver of *CBS*^+/−^ mice. **d** and **e** mRNA and protein expression of p62, BECN1, LC3 and Atg12 were determined by qRT-PCR and western blot respectively in the liver of *CBS*^+/−^ mice after injection with lentivirus expressing *CFTR*

To know whether promoting *CFTR* expression could be an important intervention to protect liver against Hcy-induced autophagy, *CBS*^+/-^ mice were injected with lentivirus expressing *CFTR* (Lv-CFTR) (tilter = 2 × 10^7^ TU/mL), lentivirus expressing GFP (Lv-GFP) (tilter = 2 × 10^7^ TU/mL) or PBS by the tail vein, and after 30 days, mRNA and protein were isolated from livers. As Fig. 3a shows, both *CFTR* mRNA and protein expression were increased in the liver of Lv-CFTR-injected mice compared with control mice. Subsequently, we analyzed the level of aminopherase (AST and ALT). We found that aminopherase (AST and ALT) levels decrease in the sera of Lv-CFTR-injected mice as compared with mice injected with Lv-GFP (Fig. 3b). Meanwhile, we examined the histopathology of the livers by H&E (Fig. 3c upper) and Oil Red O staining (Fig. 3c middle). Lv-GFP-injected mice had inflammatory cell infiltrates surrounding the portal and central veins, and extensive liver damage, hepatic steatosis. By contrast, livers from most Lv-CFTR-injected mice demonstrated significant attenuation of all of these pathological changes.

Further experiment was performed to investigate the relationship between *CFTR* and the hepatic autophagy, and TEM showed that autophagic vacuoles and autophagosomes reduces significantly in liver of Lv-CFTR-injected mice (Fig. 3c under). In addition, we analyzed the expression of autophagy related protein (p62, BECN1, LC3, and Atg12) in Lv-CFTR-injected mice models. Overexpression of *CFTR* significantly reduced the ratio of LC3-II/I and the expression of BECN1 and Atg12, increased the expression of p62 (Fig. 3d, e). All these results suggest that *CFTR* overexpression attenuated HHcy induces liver injury and autophagy in *CBS*^+/-^ mice.

### DNMT1 positively regulates *CFTR* promoter methylation in Hcy-treated hepatocytes

**Fig. 4:**
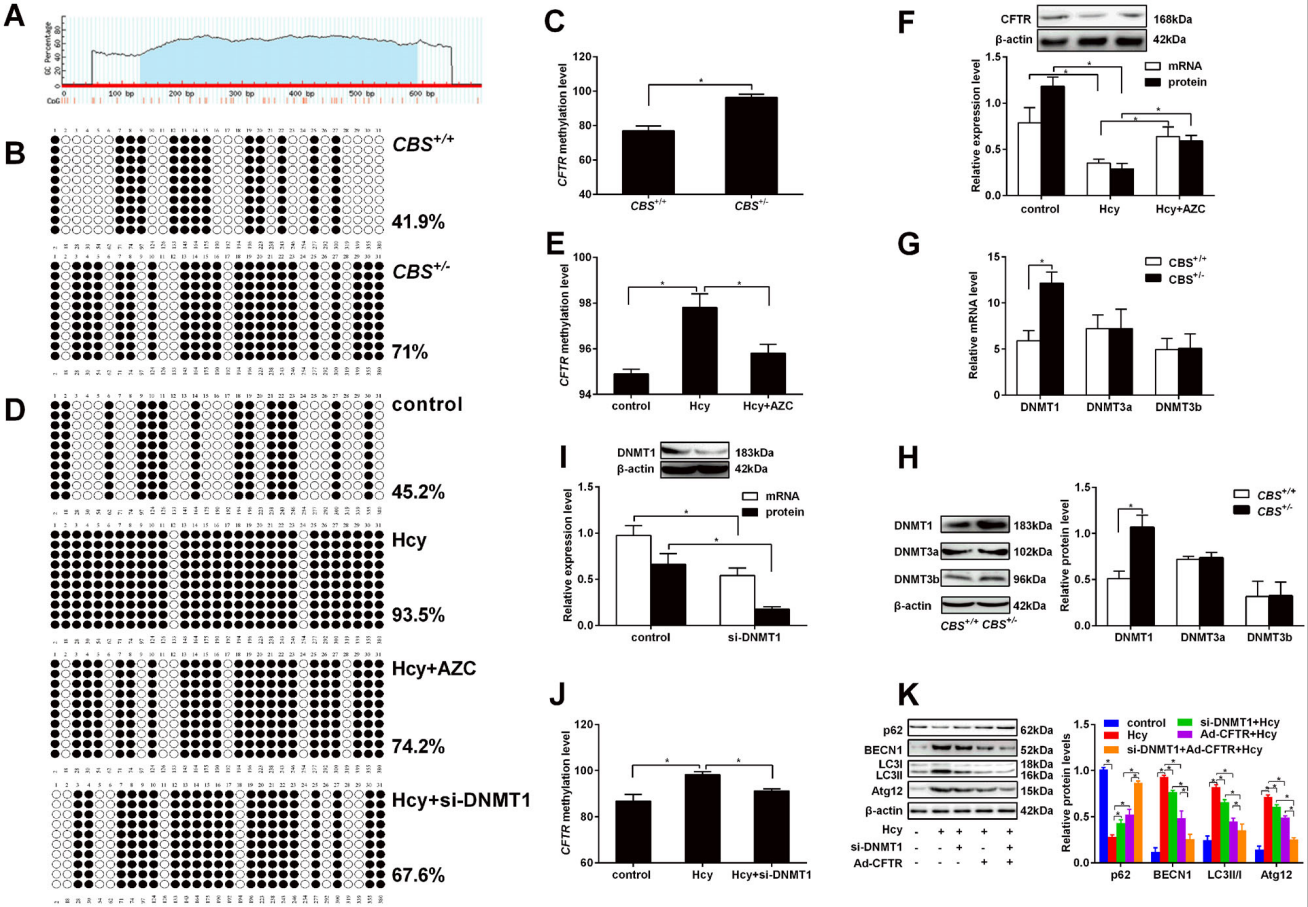
Hcy induces liver autophagy by downregulation of *CFTR* expression via DNA methylation. Eight to 10 weeks old cystathionine b-synthase (*CBS*) heterozygous knockout mice (*CBS*^+/−^) were fed with regular mice chow and water ad libitum. Human hepatocytes HL-7702 were treated with L-Hcy and adenovirus harboring DNMT1 small interfering RNA (si-DNMT1) respectively for 24 h. **a** A schematic drawing of the putative CpG islands in the 5’-flank region of *CFTR*. (B, C) The total methylation rate of CpG rich region (−572 bp/−262 bp) within the proximal promoter of the *CFTR* gene was detected by bisulfite-sequencing PCR (BSP) method in liver of *CBS*^+/-^ mice and (**d**, **E**) HL-7702 cells. Black and white circles represent methylated and unmethylated CpGs respectively. **f** qRT-PCR and western blot were used to detect the mRNA and protein expression of *CFTR* in HL-7702 cells treated with Hcy (100 μmol/L) or Hcy (100 μmol/L) plus 5-azacytidine (AZC) (10 μmol/L). **g**, **h** The mRNA and protein expression of DNMTs in liver tissues of mice were detected by qRT-PCR and western blot. **i** The mRNA and protein expression of DNMT1 were detected by qRT-PCR and western blot in hepatic cells infected with adenovirus si-DNMT1. **j** Bisulfite sequencing detected the DNA methylation of *CFTR* in HL-7702 cells infected with the adenovirus si-DNMT1. **k** Western blot was performed to detect the protein expression of autophagy related proteins p62, BECN1, LC3 and Atg12 in hepatocytes infected with adenovirus CFTR and adenovirus si-DNMT1. Mean ± s.d. of three experiments performed in triplicate. * *P* < 0.05

To explore whether Hcy regulates *CFTR* expression through CpG methylation, CpG island (−572 bp/-262 bp) was predicted in the region of *CFTR* promoter using Methprimer software (Fig. 4a). The methylation status within CpG islands in *CFTR* promoter were detected by bisulfite sequencing PCR (BSP). As shown in Fig. 4b, c, *CFTR* DNA methylation in the liver of *CBS*^+/-^ mice is higher than that in the liver of the *CBS*^+/+^ mice. In hepatocytes, Hcy treatment enhances the DNA methylation of *CFTR* promoter as well, and the inhibition can be partly reversed by 5-azacytidine treatment (Fig. 4d, e). Accordingly, these results were associated with a low expression of *CFTR* mRNA and protein (Fig. 4f).

To further know the role of DNMTs in promoter methylation of *CFTR* both in vivo and in vitro, we examined expression of DNMT1, 3a and 3b. The results indicated that *DNMT1* mRNA expression is elevated in the liver of *CBS*^+/-^ mice, which is consistent with protein quantification results (*P < *0.01, Fig. 4g, h). On the contrary, there is almost no difference in the expression of DNMT3a and DNMT3b in the liver between *CBS*^+/-^ mice and *CBS*^+/+^ mice. To further investigate whether DNMT1 regulates hypermethylation of *CFTR* promoter, we knocked down DNMT1 expression by adenovirus harboring DNMT1 small interfering RNA (si-DNMT1) in HL-7702 cells (Fig. 4i). Adenovirus si-DNMT1 can alleviate the upregulation of DNA methylation in the regions of CpG island of *CFTR* in Hcy-treated HL-7702 cells (Fig. 4d, j). Next, to verify whether DNMT1 could promote autophagy through downregulated *CFTR* expression in hepatocytes, we co-infected Ad-CFTR and si-DNMT1 into hepatocytes pre-treated by Hcy. The result showed that the ratio of LC3-II/I and the expression of BECN1, and Atg12 decreased and the expression of p62 increased in hepatocytes co-infected with Ad-CFTR and si-DNMT1 (Fig. 4k). Taken together, these data indicate that DNMT1 hypermethylates *CFTR* promoter enhances the level of autophagy in Hcy-treated hepatocytes.

### Hcy-induced elevation of EZH2 is responsible for *CFTR* promoter H3K27me3

**Fig. 5:**
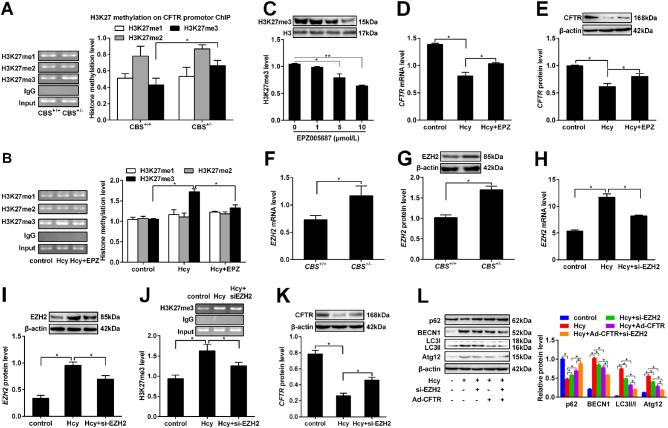
Hcy-induced elevation of EZH2 is responsible for *CFTR* promoter H3K27me3. Eight to 10 weeks old cystathionine b-synthase (*CBS*) heterozygous knockout mice (*CBS*^+/−^) were fed with regular mice chow and water ad libitum. Human hepatocytes HL-7702 were treated with L-Hcy and adenovirus harboring EZH2 small interfering RNA (si-EZH2) respectively for 24 h. **a** The H3K27me1, 2, 3 levels on the *CFTR* promoter were examined in liver of mice by ChIP-PCR. **b** The levels of H3K27me3 on the *CFTR* promoter were examined by ChIP-PCR in hepatocytes treated with 100μmol/L Hcy and 100 μmol/L Hcy plus 10 μmol/L EPZ005687. **c** The H3K27me3 levels in the *CFTR* promoter of hepatocytes treated with different concentration of agonist of H3K27me3 (1, 5, 10 μmol/L EPZ005687). **d**, **e** qRT-PCR and western blot were used to detect the mRNA and protein expression of *CFTR* in the hepatic cells treated with 10 μmol/L EPZ005687 and 100 μmol/L Hcy plus 10 μmol/L EPZ005687. **f**,** g** The mRNA and protein expression of EZH2 were detected by qRT-PCR and western blot in HL-7702 cells exposed to 100μmol/L Hcy for 24 h. (**H**-**I**) The mRNA and protein expression of EZH2 were detected by qRT-PCR and western blot in hepatic cells infected with adenovirus si-EZH2. (**J**) The level of H3K27me3 on *CFTR* promoter were detected by ChIP-PCR in hepatic cells infected with adenovirus si-EZH2. (**K**) The mRNA and protein expression of *CFTR* were detected by western blot in hepatic cells infected with adenovirus si-EZH2. (**L**) The protein expression of autophagy related protein factors p62, BECN1, LC3 and Atg12 were detected by western blot in hepatocytes infected with adenovirus (Ad-CFTR, si-EZH2, Ad-CFTR and si-EZH2). Mean ± SD of three experiments performed in triplicate. * *P* < 0.05

To study whether histone H3 lysine 27 (H3K27) methylation regulates *CFTR* expression, we detected H3K27 methylation on *CFTR* promoter by ChIP-PCR. The results demonstrated trimethylated H3K27 (H3K27me3) in the proximal promoter region of *CFTR* in the liver of *CBS*^+/-^ mice, but not H3K27me1 and H3K27me2, is higher than that in the liver of *CBS*^+/+^ mice (Fig. 5a). Meanwhile, similar results were also observed at the cell level, with increased level of H3K27me3 detected in the hepatocytes treated with Hcy (Fig. 5b). To check the inhibiting effect of EPZ005687 on H3K27me3, we detected the level of H3K27me3 in hepatocytes after treatment with different concentration EPZ005687. Western blot showed that downregulation of H3K27me3 level is positively correlated with the concentration of the EPZ005687 (Fig. 5c). Furthermore, Hcy treatment of HL-7702 cells inhibited *CFTR* expression as well, and the downregulation can be partly reversed by EPZ005687 treatment. (Fig. 5d, e), suggesting a possible role of H3K27me3 in the regulation of *CFTR* expression in Hcy treated liver.

We also tested impact of Hcy on the expression of EZH2 and H3K27me3 in *CFTR* promoter. The results showed that protein expression of EZH2 was enhanced in the liver of *CBS*^+/-^ mice (Fig. 5f, g) and HL-7702 cells treated with Hcy (Fig. 5h, i). Knockdown of EZH2 by adenovirus harboring EZH2 small interfering RNA (si-EZH2) in HL-7702 cells inhibited H3K27me3 in *CFTR* promoter (Fig. 5j) and these results were associated with a high expression of *CFTR* protein (Fig. 5k). The result suggests that Hcy downregulates the expression of *CFTR* through enhanced *CFTR* promoter H3K27me3 by EZH2.

Based on the results above, whether EZH2 could promote autophagy in hepatocytes was further studied. The result showed that the ratio of LC3-II/I and the expression of autophagy-related proteins BECN1 and Atg12 decreased and the expression of p62 increased in hepatocytes co-infected with Ad-CFTR and si-EZH2 (Fig. 5l). Taken together, these data indicate that EZH2 hypermethylates *CFTR* promoter H3K27me3 enhances the level of autophagy in Hcy-treated hepatocytes.

### *CFTR* promoter DNA methylation and histone methylation interact with each other in hepatocyte autophagy induced by Hcy

**Fig. 6:**
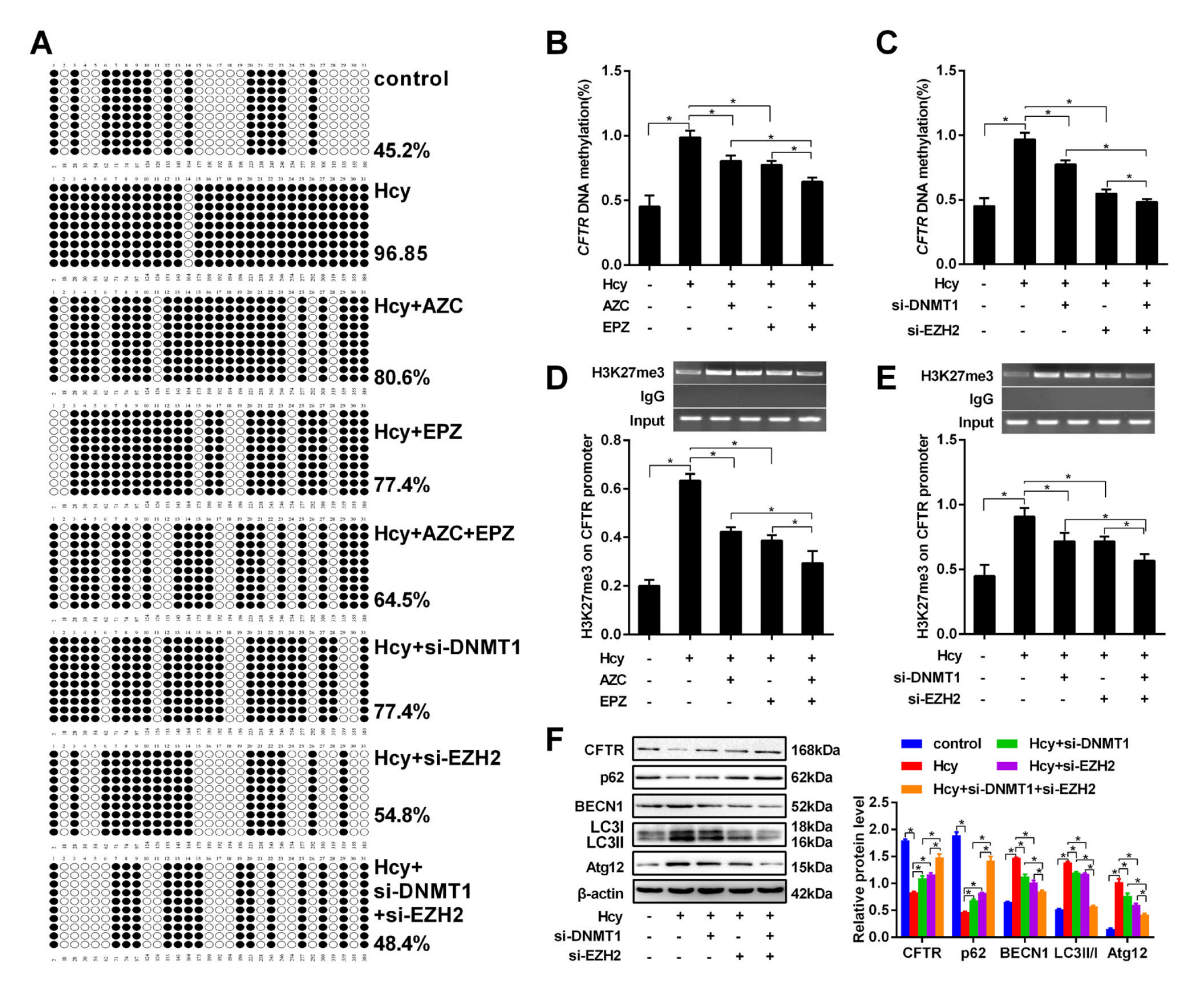
Interaction between DNA methylation and histone methylation in *CFTR* promoter. Human hepatocytes HL-7702 were treated with Hcy or infected with adenovirus vector (si-DNMT1 and si-EZH2) for 24 h. **a**–**c** The DNA methylation of *CFTR* was detected by BSP after the hepatocytes were treated with 5-azacytidine, an inhibitor of DNA methyltransferase, and EPZ005687 (EPZ), an inhibitor of histone lysine methyltransferase, or infected with adenovirus vector EZH2 RNAi (si-EZH2) and adenovirus vector DNMT1 RNAi (si-DNMT1). **d**, **e** ChIP-PCR were used to detect the H3K27me3 levels in the *CFTR* promoter in hepatocytes treated with 5-azacytidine, an inhibitor of DNA methyltransferase, and EPZ005687 (EPZ), an inhibitor of histone lysine methyltransferase or infected with si-EZH2 and si-DNMT1. **f** The protein levels of *CFTR* and autophagy-related protein factors p62, BECN1, LC3, and Atg12 were detected by western blot in hepatocytes treated with si-EZH2 and si-DNMT1. Mean ± s.d. of three experiments performed in triplicate. * *P* < 0.05

As an inhibitor of histone lysine methyltransferase that can inhibit histone methylation, EPZ005687 inhibited DNA methylation of *CFTR* promoter in addition to the inhibition of histone methylation in HL-7702 cells (Fig. 6a, b, d). Meanwhile, DNA methyltransferase inhibitor 5-azacytidine (AZC) can inhibit H3K27me3 of *CFTR* promoter in addition to the inhibition of DNA methylation of *CTFR* promoter (Fig. 6a, b, d), suggesting that DNA methylation and histone methylation of *CTFR* promoter could correlate with each other. To further confirm that, EZH2 and DNMT1 that mediate the histone methylation and DNA methylation in hepatocytes were knocked down respectively. The results showed that knockdown of either EZH2 or DNMT1 can repress both DNA methylation and histone methylation (Fig. 6c, e). To elucidate these effects on the regulation of autophagy-related protein expression in hepatocytes, we perform western blot and found that the ratio of LC3-II/I and the expression of autophagy-related proteins BECN1 and Atg12 decreased and the expression of p62 and *CFTR* increased in hepatocytes co-infected with si-EZH2 and si-DNMT1 (Fig. 6f). All these data suggest that DNA methylation and histone methylation interaction may play a vital role in *CFTR* downregulation, which further mediates Hcy-induced hepatic autophagy.

## Discussion

In the current study, we examined the biological effects of elevated Hcy and the involvement of *CFTR* in Hcy-induced autophagy. Our findings proved a cross-talk between DNA methylation and histone methylation in the regulation of *CFTR* expression, leading to hepatic autophagy induced by Hcy. The results provide fundamental insight into how autophagy homeostasis is maintained.

Dysfunction of liver alters methionine metabolism, resulting in elevated Hcy that is released into the plasma^22^. In return, Hcy can influence the status of liver as well, and autophagy has been proposed to explain the pathogenic effects of Hcy in hepatic injury^23^. In this study, we found that autophagy occurrs in *CBS*^+/-^ mice liver. Additionally, similar results were found in Hcy-treated hepatocytes. The recent discovery of Hcy-triggered autophagy contributes to brain neuronal cell injury following focal cerebral ischemia-reperfusion with the generation of autophagosomes and the upregulation of LC3B/BECN1 protein expression in neurons^24^. The possible explanation is that the oxidative damage-mediated autophagy may be a molecular mechanism underlying hepatocellular damage by elevated Hcy level, which further confirms our results that Hcy promotes autophagy to induce liver injury.

Recent study reported that *CFTR* orchestrates a proteostatic network that influences multiple cellular functions including autophagy by acting as a hub protein^25,26^. Our results demonstrated that *CFTR* expression decreases in *CBS*^+/-^ mice, which is consistent with that *CFTR* expression in mice is downregulated during a much later stage of tunicamycin-induced autophagy. Then the expression of p62, BECN1, LC3 and Atg12 were measured in *CFTR*-overexpressed hepatocytes. Results of the present study revealed that, in the presence of Hcy, autophagy was enhanced when transcriptional activation of *CFTR* was reduced in hepatocytes. Overexpression of *CFTR* inhibited the formation of autophagosomes and the expression of autophagy-related proteins BECN1, LC3-II/I, and Atg12, while the expression of p62 increased in Hcy-treated hepatocytes and *CBS*^+/-^ mice injected with lentivirus expressing *CFTR*. A previous study provided striking molecular evidence that etiologic *CFTR* mutations trigger a cascade of events culminating in the autophagy-related protein BECN1 depletion and impairment caused by autophagy^27^. Additionally, Tosco A et al.^28^ found that correct of dysfunctional *CFTR* may be the most intellectually satisfying way for therapeutic strategy in cystic fibrosis, by restore autophagy, all which support our observations that *CFTR* plays a crucial role in hepatocyte autophagy. One of the traditional metabolic pathways of Hcy is sulfur transfer way, and *CFTR* has the ability to form disulfide bond. It has been recently reported that Hcy may transfer sulfhydryl group to other substances (such as *CFTR*) to cause hepatic autophagy, which might be a novel mechanism for Hcy-induced liver injury. To our confusion, Hcy is a non-essential sulfhydryl-containing amino acid derived from methionine metabolism, it is dynamically regulated by re-methylation and trans-sulfuration pathway. It is not comprehensive to explain the pathogenic mechanism of Hcy only by means of trans-sulfuration pathway, which needs to be further explored.

Elevated levels of Hcy can alter global DNA methylation and induce promoter specific methylation in many genes implicated in human diseases including cancer, neurodegenerative diseases and cardiovascular disease^29–31^. Earlier reports^32^ have demonstrated that Hcy triggers inflammatory responses in macrophages through inhibiting CSE-H_2_S signaling via DNA hypermethylation of CSE promoter. Our previous work also indicated that DNA hypermethylation suppresses p53 expression in cardiac tissue of *ApoE*^−^^*/*−^ mice with HHcy^33^. In present work, we found that DNA hypermethylation represses *CFTR* transcription in *CBS*^+/−^ mice liver which has high levels of Hcy. Furthermore, *CFTR* DNA methylation level was increased in hepatocytes co-incubated with Hcy. We also found an increase of DNMT1 expression both in *CBS*^+/−^ mice and hepatocytes. Since Hcy is a precursor of *S*-adenosyl methionine (SAM) which is a universal methyl donor, high level of Hcy can lead to high level of SAM. In the meantime, SAM is one of the substrates for DNMTs, high levels of SAM increase the activity of DNMTs, which is reflected in the global methylation experiment. Apart from DNA methylation, Hcy is associated with histone methylation, which alters the expression of remodeling genes^34^. Our study showed an increase in H3K27me3 on *CFTR* promoter and the expression of EZH2 in *CBS*^+/-^ mice. Accordingly, treating hepatocytes with Hcy resulted in promoted H3K27me3, *CFTR* hypermethylation, and silencing of *CFTR*. To explicit the relationship between EZH2 and DNMT1, si-DNMT1 and si-EZH2 were co-transfected into hepatocytes pre-treated by Hcy, we found downregulated *CFTR* DNA methylation, decreased H3K27me3 and increased expression of *CFTR*. These results suggested that interaction between EZH2 and DNMT1 downregulates *CFTR* expression in hepatocytes treated with Hcy. There are a few mechanisms supporting our findings. First, methylation of DNA and histone use a common methyl donor (i.e., SAM). In addition, modifications of DNA and histone methylation are carefully to modulate gene expression programming in the organism through direct interactions between histone and DNMTs. Alterations in intracellular availability of SAM can result in changes in gene expression. Furthermore, many studies suggested that Hcy can also directly affect the epigenetic regulation of gene expression^35^.

In conclusion, our data demonstrated a crucial role of *CFTR* in reducing hepatic autophagy in the presence of Hcy. We also revealed that *CFTR* may mediate the effects of metabolic perturbations of Hcy-induced autophagy. DNA methylation and histone methylation regulate *CFTR* expression leading to autophagy induced by Hcy is a novel mechanism of liver injury. However, the underlying mechanism of *CFTR* in Hcy-induced hepatic autophagy needs to be further explored.

## Materials and methods

### Animals

All animal experiments were approved and carried out in accordance with the Institutional Animal Care and Use Committee of the University of Ningxia Medical University. Eight to 10 weeks old cystathionine beta-synthase (*CBS*) heterozygous knockout mice (*CBS*^+/−^) were obtained from Jackson Laboratory (Bar Harbor, ME) and maintained in the animal facility center at University of Ningxia Medical University. The mice were fed with regular mice chow and water ad libitum. Mice genotypes were determined by a PCR of DNA obtained from tail biopsies with a specific set of primers^36^. For evaluation of the therapeutic potential of recombinant lentiviruses expressing human *CFTR* (Lv-CFTR), 19 mice were injected with Lv-CFTR and Lv-GFP into the tail vein respectively, the titers averaged 2 × 10^7^ TU/mL, and total volume was 80 mL. Equal volume of PBS was used as a vehicle control. Thirty days after lentivirus injection, mice were sacrificed and livers were flash-frozen in liquid N_2_ and processed to obtain total protein.

### Liver tissue preparation and morphologic observation

Thirty days after treatment, all mice were fasted but supplied with water for 24 h. Then, mice peritoneally injected with 3% pentobarbital sodium according to injection dose to mouse weight of 2 mL/kg. Abdominal cavity of anesthetic mouse was incised, and then inferior vena cava blood was collected using 10 mL syringe. After standing for 3 h at 4 °C, blood samples were centrifuged at 5000 × *g* for 15 min. Supernatant was saved at −80 °C for future use. Liver was incised and two segments (1.0 cm × 1.0 cm × 0.3 cm) were cut from right lobe of the liver. Two liver segments were fixed in 10 % neutral formalin and embedded in optimum cutting temperature compound (OCT). The remained liver samples were saved at −80 °C for future use. To observe morphologic changes in liver tissues, the HE staining and red O staining of liver segments was performed according to previous description^37,38^.

### Plasmatic parameters estimation

The plasmatic alanine aminotransferase (ALT) and aspartate transaminase (AST) activities were measured using the Cobas e411 analyzer (Roche Diagnostics, Mannheim, Germany), according to the controls and calibration of the Cobas e411 analyzer (Roche Diagnostics, Mannheim, Germany).

### Transmission electron microscopy (TEM)

The livers were fixed in 0.1 M cacodylate buffer containing 2.5% glutaraldehyde at 4 °C for at least 24 h. The livers were then dissected into quadrants, osmicated, washed, block contrasted with 2% aqueous uranyl acetate, dehydrated through a graded series of ethanol, and embedded in Epon 812 (Plano GmbH, Marburg, Germany). Ultrathin sections (about 50 nm) were mounted on pioloform-coated slot copper grids and contrasted with uranyl acetate (5 min) and lead citrate (3 min). The specimens were examined using a Zeiss EM 902 transmission electron microscope (Zeiss, Oberkochen, Germany) at 80 kV. Photographs were taken by a CCD camera (Proscan, Lagerlechfeld, Germany).

### Cell culture and virus transfection

Human hepatocytes (HL-7702) were obtained from the Japanese Collection of Research Bioresources and cultured in RPMI-1640 medium (Thermo, USA) containing 10% fetal bovine serum (FBS) (Hyclone, USA) and 1% penicillin (Thermo, Waltham, MA, USA) humidified with 5% CO_2_ air at 37 °C. The cells were transfected with Ad-CFTR when they were 80% confluent. The control cells were transfected with Ad-GFP. After 48 h, the transfection efficiency was detected by fluorescence microscope, and then the cells were treated with L-Hcy (100 μmol/L) (Sigma, USA) for another 24 h for further experiments.

### Quantitative real-time PCR

Quantitative real-time PCR (qRT-PCR) for *CFTR*, *DNMT1* and *EZH2* genes was performed using an FTC3000 qRT-PCR detection system as described previously^17,39^. qRT-PCR kits were from Takara Biotechnology Co., Ltd. (Dalian, China). The first-strand cDNA reaction (0.5 μL) was subjected to qRT-PCR amplification using gene-specific primers (Table 1). All primers were synthesized by Sangon Biotech Co., Ltd. (Shanghai, China). qRT-PCR reaction was performed with an initial denaturation at 95 °C for 10 min followed by 40 cycles of denaturation at 95 °C for 20 s, annealing at 58 °C for 30 s, and extension at 72 °C for 30 s, and, for a final step, a melting curve of 94 °C for 90 s, 60 °C for 180 s, and 94 °C for 10 s. Glyceraldehyde phosphate dehydrogenase (GAPDH) was used as the invariant control; mRNA expression was determined by 2^−ΔΔCT^ method. Results were obtained from triplicate experiments.

**Table 1:** Primer sequences for qRT-PCR

Gene	Species	Primer Sequence (5′ → 3′)	Length (bp)	Annealing Temperature (°C)
*CFTR*	Human	F: AGAACTGAAACTGACTCGGAAGGR: GCAGAATGAGATGGTGGTGAA	157	59.0
	Mouse	F: ACAGGATAGAAGCGATGTTGGATTGCR: GAGGCTTGTGCTTGCTGGAGTG	177	58.5
*DNMT1*	Human	F: GCGGCAGACCATCAGGCATTCR: CGTTCTCCTTGTCTTCTCTGTCATCC	140	60.0
	Mouse	F: TCACTTGGACGAGGACGAGGACR: TACCTGCTCTGGCTCTGCTTCC	115	57.5
*DNMT3a*	Human	F: GGAGCCACCAGAAGAAGAGAAGAATCR: TGCCGCACCTCGTACACCAG	189	57.5
	Mouse	F: AACTGCTTCTTGGAGTGTGCTTACCR: GGTCTTCCTTAATGGCTGCCTGAG	184	59.0
*DNMT3b*	Human	F: AGCTCTTACCTTACCATCGACCTCACR: TACTCTGAACTGTCTCCATCTCCACTG	164	57.5
	Mouse	F: GAAGGTGCGTCGTTCAGACAGTAGR: TCAGAAGCAGCAGAGTCATTGGTTG	93	58.0
*EZH2*	Human	F: CATTCGGTAAATCCAAACTGCR: CGACATACTTCAGGGCATCA	148	59.0
	Mouse	F: AGAGTGGAAGCAGCGGAGGATACR: CATTATAGGCACCGAGGCGACTG	157	56.5
*p62*	Human	F: GCTGTGGATGAAGTGGAACCTCTACR: CTGGCTGGAAGTCAGGCTGTAAC	109	56.8
	Mouse	F: ACATACGCAGAACAGAGTTACGAAGGR: CATTCCAGTCATCTTGTCCGTAGGC	133	58.0
*BECN1*	Human	F: ATGGAAGGGTCTAAGACGTCCAACAR: CGCCTGGGCTGTGGTAAGT	156	55.7
	Mouse	F: GACGAACTCAAGAGTGTGGAGAACCR: AGATGTGGAAGGTGGCATTGAAGAC	97	58
*LC3B*	Human	F: ATGAAGATGAGATTCTTCAGTTCTCR: TCGTCTTTCTCCTGCTCGTAG	326	57.4
	Mouse	F: GCTGGTGCTGCCTATGTTGTCTCR: AATACATGGTCCTGTGGCAAGATTCC	146	56.5
*Atg12*	Human	F: ATGACTAGCCGGGAACACCAAGTTTR: TCCTCCGCCATCTTGCTTG	152	56.5
	Mouse	F: TGCTGAAGGCTGTAGGAGACACTCR: TGATGAAGTCAATGAGTCCTTGGATGG	93	57.0
*GAPDH*	Human	F: CGGATTTGGTCGTATTGGGR: CGCTCCTGGAAGATGGTGAT	213	56
	Mouse	F: CCCTTAAGAGGGATGCTGCCR: ACTGTGCCGTTGAATTTGCC	263	55.0

*F* forward primer; *R* reverse primer

### Western blot

Western blot analysis was performed as previously described^17,39^. Murine liver tissues and HL-7702 cells were lysed in a lysis buffer (KeyGEN, China) containing the protease inhibitor phenylmethanesulfonyl fluoride (PMSF, KeyGEN, China) at 4 °C for 30 min followed by centrifugation to remove cell debris. Protein concentration was measured using BCA protein assay kit (Beyotime Institute of Biotechnology). The protein was boiled and subjected to western blot with antibodies against CFTR, DNMT1, DNMT3a, DNMT3b, EZH2, p62, BECN1, microtubule-associated protein 1 light chain 3 (LC3), Atg12 and β-actin (all from Abcam Inc., Cambridge, MA, USA) respectively. Optical densities of the bands were analysed with Bio-Rad image analysis (Bio-Rad, Hercules, CA, USA).

### Cell viability assay

Cell viability was determined by MTT assay as originally described by Mosmann et al.^40^. In brief, hepatic cells were plated on a 96 well plate at a density of 10^5^ cells/well and incubated with different concentrations of Hcy (0–500 μM) in serum-free RPMI-1640 at 37 °C for 24 h. Then MTT was added to each well followed by incubation for 4 h at 37 °C. The medium was removed and wells were rinsed twice with PBS. After that, 100 μl of DMSO was added to each well to dissolve the cell membrane and bring formazan into the solution. The amount of formazan was then measured by collecting absorbance values at 570 nm by spectramax3000 spectrophotometer (Molecular Devices, Sunnyvale, CA).

### Autophagy detection using mRFP-GFP adenoviral vector

mRFP-GFP-LC3 adenoviral vectors were purchased from HanBio Technology Co. Ltd. (HanBio, shanghai, China). Hepatocytes were seeded in six-well plates andthey were incubated in growth medium with the adenoviruses at a MOI of 100 for 2 h at 37 °C, and were then grew in medium with addition of 100 μmol/L Hcy for another 24 h at 37 °C. Autophagy was observed under a confocal fluorescence microscope (Olympus BX 51, Tokyo, Japan). Autophagic flux was determined by evaluating the number of GFP and mRFP puncta (puncta/cell were counted).

### Chromatin immunoprecipitation (ChIP) assays

Formaldehyde (1%) was added to the samples to cross-link protein–DNA complexes. ChIP trials were conducted using a ChIP assay kit (Merck Millipore, DA, Germany). After cross-linking, the DNA was fragmented by sonication (UCD-200; Diagenode, Liège, Belgium), which consisted of 25 cycles of 30 s each with an interval of 30 s to cool down. Then, protein–DNA complexes were precipitated with an anti-H3K27me1, H3K27me2 and H3K27me3 (Abcam, MA, USA) antibody. DNA was extracted using a DNA purification kit (Merck Millipore). The experiment contained both a positive control group (precipitated with an anti-RNA polymerase II antibody) and a negative control group (precipitated with normal mouse IgG). The amount of extracted DNA was determined by qRT-PCR. The primers were designed as follows: the forward primer is: 5’-CGACGGACGATCTATGATGC-3’ and the reverse primer is: 5′-AGTCGCAGCTTCTCGTCAG-3′.

### Bisulfite sequencing

DNA was extracted from the cells using a DNA extraction kit (AP-MN-MS-GDNA-250; Axygen, CA, USA) and then treated with bisulphate (ZYMO RESEARCH). The PCR products were gel extracted (AP-GX-250; Axygen) and ligated into a plasmid vector using the pMD18-T Vector (D101B; Takara). Plasmid transformed DH5a bacteria were cultured overnight, and the plasmid DNA was isolated (Axygen). At least 10 separate clones were chosen for sequence analysis.

### RNA interference

Oligonucleotide sequences for RNA interference (RNAi) controls (nonsilencing), DNMT1 and EZH2 were as following: nonsilencing small interfering RNA (siRNA), UUCUCCGAACGUGUCACGU; DNMT1 siRNA, CACUGGUUCUGCGCUGGGA; EZH2 siRNA, AGCUCUUUGACGUGCAAUA. All RNAi oligonucleotides were purchased from Shanghai GeneChem Company (Shanghai) and transfected into HL-7702 cells using the Lipofectamine 2000 transfection kit (Invitrogen, Carlsbad, CA) according to the manufacturer’s instructions.

### Statistical analysis

Each experiment was performed 3 times. Single parameters between 2 groups was compared by the paired Student’s *t-*test. One-way ANOVA was used to compare multiple groups, followed by Newman–Keuls test. *P*-values < 0.05 are considered as statistically significant.
